# Effect of *N*-methyl-d-aspartate receptor enhancing agents on cognition in dementia: an exploratory systematic review and meta-analysis of randomized controlled trials

**DOI:** 10.1038/s41598-021-02040-5

**Published:** 2021-11-26

**Authors:** Chun-Hung Chang, Chieh-Yu Liu, Shaw-Ji Chen, Hsin-Chi Tsai

**Affiliations:** 1 Institute of Clinical Medical Science, China Medical University, Taichung, Taiwan, ROC; 2 Department of Psychiatry and Brain Disease Research Center, China Medical University Hospital, Taichung, Taiwan, ROC; 3 An Nan Hospital, China Medical University, Tainan, Taiwan, ROC; 4 Biostatistics Consultant Lab, Department of Health Care Management, National Taipei University of Nursing and Health Sciences, Taipei, Taiwan, ROC; 5 Department of Psychiatry, Taitung MacKay Memorial Hospital, Taitung, Taiwan, ROC; 6 Department of Medicine, Mackay Medical College, New Taipei, Taiwan, ROC; 7 Department of Psychiatry, Tzu-Chi General Hospital, Hualien City, Taiwan, ROC; 8 Institute of Medical Science, Tzu-Chi University, No. 707, Sec. 3, Chung Yang Rd., Hualien 970, Taiwan, ROC

**Keywords:** Diseases, Medical research, Molecular medicine, Neurology

## Abstract

Multiple *N*-methyl-d-aspartate (NMDA) receptor enhancing agents have had promising effects on cognition among patients with dementia. However, the results remain inconsistent. This exploratory meta-analysis investigated the effectiveness of NMDA receptor enhancing agents for cognitive function. PubMed, the Cochrane Central Register of Controlled Trials, and the Cochrane Database of Systematic Reviews were searched for randomized controlled trials (RCTs). Controlled trials assessing add-on NMDA receptor enhancing agent treatment in patients with dementia and using cognition rating scales were eligible and pooled using a random-effect model for comparisons. The standardized mean difference (SMD) was calculated in each study from the effect size; positive values indicated that NMDA receptor enhancing agent treatment improved cognitive function. Funnel plots and the I2 statistic were evaluated for statistical heterogeneity. Moderators were evaluated using meta-regression. We identified 14 RCTs with 2224 participants meeting the inclusion criteria. Add-on NMDA receptor enhancing agents had small positive significant effects on overall cognitive function among patients with dementia (SMD = 0.1002, 95% CI 0.0105–0.1900, *P* = 0.02860). Subgroup meta-analysis showed patients with Alzheimer’s Disease and trials using the Alzheimer Disease Assessment Scale-cognitive subscale as the primary outcome had small positive significant effects (SMD = 0.1042, 95% CI 0.0076–0.2007, *P* = 0.03451; SMD = 0.1267, 95% CI 0.0145–0.2388, *P* = 0.2686). This exploratory meta-analysis showed a very small, positive, and significant effect on overall cognition function in patients with dementia. Studies with larger samples are needed to evaluate different cognitive domains and phases of dementia.

## Introduction

Dementia is a progressive neuropsychiatric disorder that affects memory and other cognitive functions, thus interfering with life function^1^. Alzheimer disease (AD) is the leading cause of dementia among elderly people^2,3^, and the prevalence of dementia due to AD is 4.02% among adults aged over 60 years^4^. Approximately 35.6 million adults worldwide developed dementia in 2010, and the number is projected to increase to 65.7 million in 2030^5^. Worldwide spending on AD was approximately 422 billion US dollars in 2009^6^. The main pharmacologic treatments for patients with AD are cholinesterase inhibitors and the *N*-methyl-d-aspartate receptor (NMDAR) partial antagonist memantine. However, these treatments have adverse effects, and the response is unsatisfactory^7^. Mild cognitive impairment (MCI), characterized by objective cognitive impairment, is defined as the predementia stage on the continuum of cognitive decline^8,9^. Several studies have reported that patients with MCI generally develop AD^10,11^. However, no treatment has yet been approved for MCI. Therefore, the development of alternative medications is urgently required for AD and MCI.

NMDAR has been demonstrated to play a critical role in controlling synaptic plasticity and memory function^12^. Studies have reported that overactivation of NMDAR may cause neurotoxicity, especially in the late phase of AD^13,14^. NMDAR antagonists have been developed for the treatment of AD, and memantine, an uncompetitive NMDAR partial antagonist, has been approved as an antidementia medication for moderate to severe AD^15,16^; memantine is not approved for mild AD and MCI because of unsatisfactory efficacy^17^. However, NMDAR antagonists, such as ketamine, have been demonstrated to impair spatial learning and verbal information ability in healthy humans^18^. Moreover, human studies have reported an age-related decrease in the density of NMDARs in the cerebral cortex and hippocampus^19^. These findings implicate the hypoactivation of NMDAR in cognitive impairment.

Several trials of NMDAR enhancing agents have been performed to evaluate their effect on cognitive impairment among patients with dementia. Sodium benzoate, an inhibitor of D-amino acids oxidase, can increase d-serine and thus enhance NMDAR activation^20,21^. A randomized, double-blind, placebo-controlled trial of 60 patients with early-phase AD or MCI reported a larger improvement in the AD Assessment Scale-cognitive subscale (ADAS-cog) score compared with placebo^22^. D-cycloserine, a partial agonist at the glycine site of NMDAR, has been reported to improve ADAS-cog score in patients with AD^23^. However, other trials with D-cycloserine did not report significant improvement in cognition^24,25^. Therefore, we conducted a meta-analysis on the cognitive effects of NMDAR enhancing agents in dementia.

## Methods

### Search strategy and inclusion criteria

**Figure 1:**
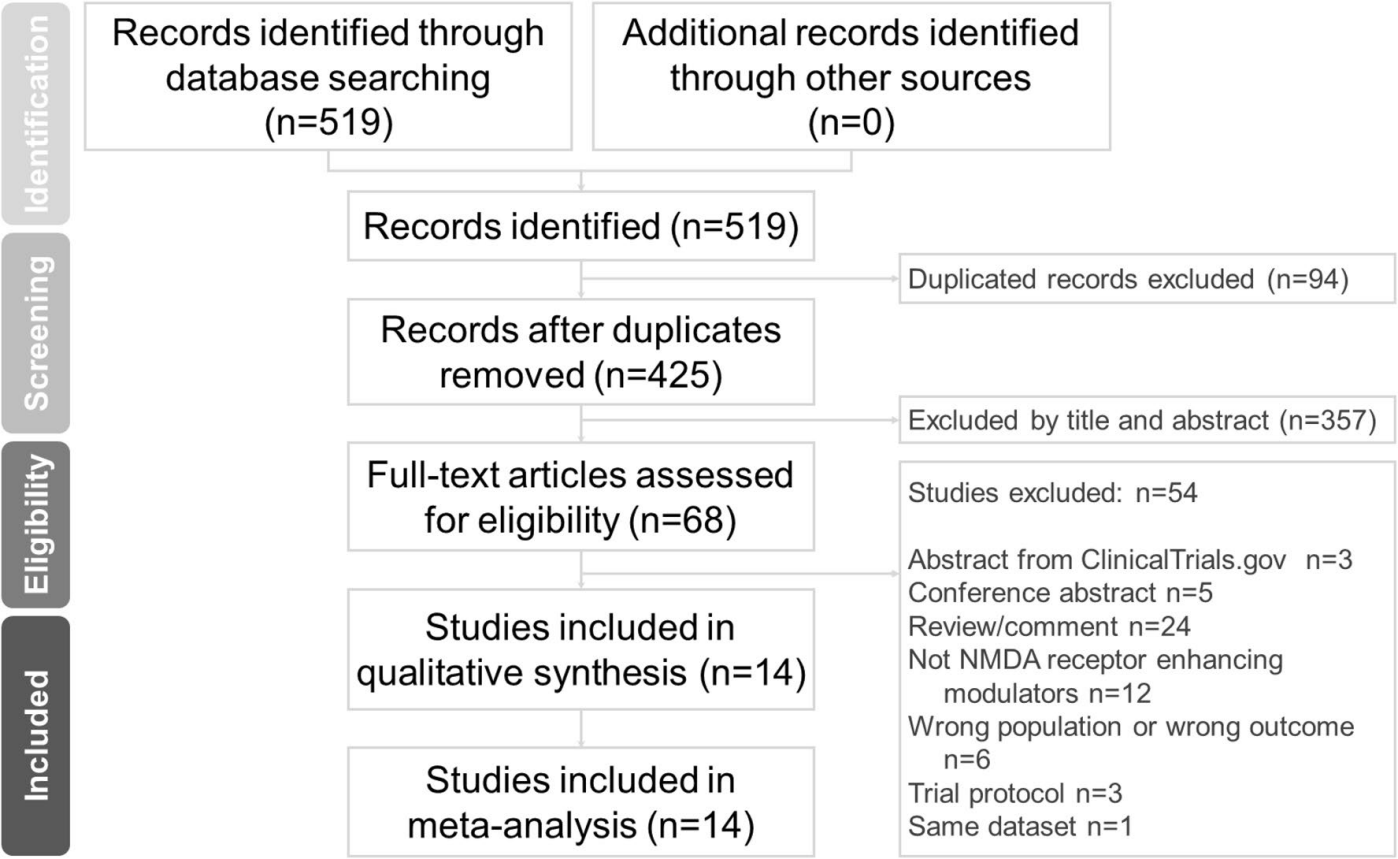
PRISMA flow diagram of the search and identification of included studies. Database: PubMed (n = 304), Cochrane Central Register of Controlled Trials (n = 208), Cochrane Database of Systematic Reviews (n = 7). Keyword: (Dementia OR Alzheimer*) AND (acetylcysteine OR α-amino-3-hydroxy-5-methyl-4-isoxazolepropionic acid OR AMPA OR benzoate OR CX516 OR D-cycloserine OR D-serine OR glutamine OR glutamate OR glutamate carboxypeptidase 2 OR GCP2 OR glycine OR glycine transporter type 1 OR GlyT1 OR glutamate receptor ionotropic kainate OR GRIK OR kynurenine aminotransferase OR KAT OR metabotropic glutamate receptor OR mGluR OR minocycline OR N-acetyl-aspartylglutamate OR NAAG OR *N*-methyl-d-aspartate OR NMDA OR pregnenolone OR sarcosine) AND controlled trial. Date: date available to August 7th, 2020.

Two independent authors (Chun-Hung Chang and Shaw-Ji Chen) performed a systematic literature search from the study’s inception until August 7th, 2021. We followed the drug keywords used by previous meta-analytic reviews about NMDA receptor positive modulators^26–29^. The search strategy comprised the following keywords: (Dementia OR Alzheimer*) AND (acetylcysteine OR α-amino-3-hydroxy-5-methyl-4-isoxazolepropionic acid OR AMPA OR benzoate OR CX516 OR D-cycloserine OR D-serine OR glutamine OR glutamate OR glutamate carboxypeptidase 2 OR GCP2 OR glycine OR glycine transporter type 1 OR GlyT1 OR glutamate receptor ionotropic kainate OR GRIK OR kynurenine aminotransferase OR KAT OR metabotropic glutamate receptor OR mGluR OR minocycline OR N-acetyl-aspartylglutamate OR NAAG OR *N*-methyl-d-aspartate OR NMDA OR pregnenolone OR sarcosine) AND controlled trial 26. The authors independently evaluated the titles and abstracts of all retrieved papers for eligibility. Full-text articles of potentially eligible trials were reviewed and compiled in a list of studies for inclusion. Disagreements between the two authors regarding the inclusion of a study were settled by a third reviewer (Professor Tsai). The Preferred Reporting Items for Systematic reviews and Meta-Analysis (PRISMA) were followed (Fig. 1, Supplementary Table S1)^30–32^.

### Eligibility criteria

The inclusion criteria were as follows: (a) studies on participants who received a diagnosis of dementia, (b) studies including randomized placebo-controlled trials, and (c) studies on the use of NMDAR enhancing agents as a monotherapy or adjunctive treatment to concomitant antidementia drugs. The exclusion criteria were as follows: (1) abstracts from ClinicalTrials.gov, (2) conference abstracts, (3) reviews and comments, (4) studies on treatment other than NMDAR enhancing modulators, (5) studies on a different population or outcome, and (6) trial protocols.

### Outcome measures

The effects of glutamate positive modulators on cognitive deficits in patients with dementia were investigated. Overall cognitive function (primary outcome) was compared between patients receiving NMDAR enhancing agents and those receiving placebo. The common cognitive measures in dementia are the Mini-Mental State Examination (MMSE)^33^ and the Alzheimer’s Disease Assessment Scale-cognitive subscale (ADAS-cog)^34^. The MMSE is a cognitive test commonly used to screen for dementia and measure cognitive impairment in older adults^35^. The MMSE total score ranges from 0 to 30 (highest to lowest level of cognitive impairment). The ADAS-cog consists of 11 tasks, and its score ranges from 0 to 70 (lowest to highest level of cognitive impairment).

### Data extraction

Two independent reviewers (CH Chang and SJ Chen) screened and identified articles. We recorded information including the first author, published year, number of participants, gender proportion, mean age, duration, NMDA receptor enhancing agents, and summary of neurocognitive measures (Table 1). We followed the PRISMA guidelines. The primary outcome was the difference in effect sizes between the NMDAR enhancing agent and control groups (calculated in terms of the standard mean difference [SMD] with the corresponding 95% confidence interval [CI] and *P* value). The primary author of a study was contacted to request the original data if these data were not available in the corresponding article. If no relevant data were reported in the article, other compatible statistical parameters (e.g., *P* value, sample size, or odds ratio) were used to estimate the effect size, following the Comprehensive Meta-Analysis manuals protocol and the Comprehensive Meta-Analysis website guide (https://www.meta-analysis.com/downloads/Meta-analysis%20Converting%20among%20effect%20sizes.pdf); the estimated effect sizes were then converted and pooled into SMDs. Variables of interest were extracted when possible, including first author, year, sample size, proportion of men, mean age, therapy duration, add-on NMDAR enhancing agents, and cognitive outcome measures.

**Table 1:** Summary of the characteristics of studies included in the meta-analysis.

Study (first author, year)	N	Gender (%male)	Mean age (years)	Duration	Add-on therapy	Diagnosis	Primary cognition outcome measures	Other cognitive measures	Mean cognitive score at baseline	Study design	Double-blind
Howard et al. (2020)	544	55.7	74.3(8.2)	24 m	Minocycline	Alzheimer disease	MMSE	NR	MMSE:26.4 ± 1.9	RCT	Yes
Bernard et al. (2019)	520	30.3	71.1(7.3)	24w	S47445	Alzheimer disease	ADAS-cog	MMSE	ADAS-cog: 23.6 ± 9.0 MMSE:19.7 ± 2.8	RCT	Yes
Lin et al. (2019)	97	45.1	75.5(7.8)	6w	Benzoate	Dementia	ADAS-cog	CDR, MMSE	ADAS-cog:29.3 ± 12.6	RCT	Yes
Kouzuki et al. (2019)	159	12.6	87.1(0.7)	12w	Monosodium L-glutamate	Dementia	TDAS	GBSS	TDAS:55.2 ± 3.6 GBSS:64.7 ± 3.9	RCT	No
Lin et al. (2014)	60	38.3	70.2(8.5)	24w	Benzoate	Alzheimer disease	ADAS-cog	CIBIC-plus, cognitive composite	ADAS-cog:15.3 ± 7.5	RCT	Yes
Tsai et al. (2014)	30	63.3	76.8(6.0)	8w	Sarcosine	Parkinson’s disease with dementia	CASI	CASI, MMSE, CDR	CASI:57.4 ± 23.9 MMSE:17.3 ± 6.7 CDR:2.2 ± 1.2	RCT	Yes
Chappell et al. (2007)	181	47.5	74.5(9.0)	11w	LY451395	Alzheimer disease	ADAS-cog	CIBIC, Trail Making Part A, Stylus Tapping Test (STT), Single Digit Modality Test (SDMT)	ADAS-cog:19.4 ± 9.0 MMSE:20.3 ± 3.3	RCT	Yes
Adair et al. (2001)	43	NR	NR	6 m	NAC	Alzheimer disease	MMSE	Four separate batteries^a^	MMSE:19.0 ± 3.7	RCT	Yes
Tsai et al. (1999)	17	64.7	72.2(7.3)	4w	D-Cycloserine	Alzheimer disease	ADAS-cog	NR	ADAS-cog:23.5 ± 9.0 MMSE:18.8 ± 5.3	RCT, crossover	Yes
Tsai et al. (1998)	10	40.0	74.7(8.5)	4w	D-Cycloserine	Alzheimer disease	ADAS-cog	MMSE	ADAS-cog:25.5 ± 2.7 MMSE:20.0 ± 5.2	RCT, crossover	Yes
Schwartz et al. (1996)	108	53.8	74.4(8.6)	10w	D-Cycloserine	Alzheimer disease	NR	MMSE, DRS, Implicit memory performance of words	NR	RCT	Yes
Mohr et al. (1995)	40	NR	NR	24w	Cycloserine	Alzheimer disease	Cognitive Drug Research Computerized Assessment System (CDR System)	Mattis Dementia Rating Scale (MDRS), CIBIC, Brief Cognitive Rating Scale (BCRS)	NR	RCT	Yes
Fakouhi et al. (1995)	403	46.1	73.6(8.0)	26w	Cycloserine	Alzheimer disease	Cognitive Drug Research (CDR) computerized test , DRS	MMSE	NR	RCT	Yes
Randolph et al. (1994)	12	70.0	65.0(8.4)	2w	D-Cycloserine	Alzheimer disease	ADAS-cog, RBAD	MMSE	MMSE:21.0 ± 3.3	RCT, crossover	Yes

*ADAS-cog* Alzheimer’s Disease Assessment Scale-cognitive subscale; *CASI* Cognitive Abilities Screening Instrument; *CDR* Clinical Dementia Rating; *CIBIC-plus* Clinician’s Interview-Based Impression of Change plus Caregiver Input; *DRS* Dementia Rating Scale; *GBSS* Gottfries–Bråne–Steen Scale; *HVLT* Hopkins Verbal Learning Task; *MMSE* Mini-Mental Status Examination; *NR* no reported; *RBAD* Repeatable Battery for the Assessment of Dementia; *TDAS* the Touch Panel-type Dementia Assessment Scale; WMS, Wechsler Memory Scale.

^a^Boston Naming Test, Gesture to Command, WMS Figure Reproduction(immediate), HVLT Recall (immediate), HVLT Recognition, Letter fluency, Category fluency, Judgment of Line Orientation.

### Quality assessment

Two authors (CH Chang and SJ Chen) evaluated the methodological quality of the included studies. The Jadad scoring system was used for randomized controlled trials (RCTs)^36^. The Jadad scoring system contains three items that assess randomization (2 points), blinding (2 points), and an account of all patients (1 point). Therefore, the score ranges from 0 to 5. A higher score indicates higher methodological quality.

### Meta-analysis procedure

A random-effects model was used for the meta-analysis^37^. The SMD with 95% CI was selected to compare the ESs of our primary outcome, rather than the difference in means, because each study was presumed to use different units. Furthermore, when the SMD value was higher than 0, the effect size was defined as indicating a positive effect in the NMDAR enhancing agent group compared with the placebo group. After data from included trials were extracted, Comprehensive Meta-Analysis software version 3 (Biostat, Englewood, NJ, USA) was used to perform meta-analyses. Statistical significance was defined as a two-tailed *P* < 0.05. Of note, the correction of multiple comparison was not made in this study due to this was an exploratory study rather than confirmatory study.

### Heterogeneity and publication bias

The I^2^ statistic was used as a measure of heterogeneity^37^, which was assessed using the Cochran Q test and corresponding *P* value^38^. Publication bias was assessed using funnel plots^39^ and Egger's regression test^40^ If publication bias was discovered, Duval and Tweedie's trim-and-fill procedure, a validated model for ES estimation, was used^41^.

### Sensitivity test

Studies were individually removed from the meta-analysis to verify that the results were not caused by outliers in the included studies.

### Subgroup meta-analysis and meta-regression

Subgroup analyses were performed when at least two independent data sets were available^41^. Samples were segmented by variables, including diagnosis of dementia, cognitive measure, study design, NMDAR enhancing agent, drug pathway in enhancing glutamatergic neurotransmission, duration of therapy, and age range. Furthermore, meta-regression analyses were performed for each potential moderator, including baseline mean ADAS-cog total score, proportion of men, and mean age.

## Results

### Characteristics of included studies and patients

The final quantitative analysis included 2224 participants from 14 trials^22–25,42–51^. The PRISMA flow diagram is displayed in Fig. 1, and the study characteristics are summarized in Table 1 and Supplementary Table S2. The mean number of subjects in each study was 158.86 ± 188.73 (range: 10–544), and the mean duration of the studies was 20.21 ± 25.69 weeks (range: 2–104 weeks). The mean age of the subjects was 74.10 ± 5.14 years. The mean proportion of men was 47.27% ± 16.02%. Nine NMDAR enhancing agents were investigated. The numbers of studies and subjects for each compound investigated were as follows: benzoate, two studies, n = 157; cycloserine, two studies, n = 443; D-cycloserine, four studies, n = 147; LY451395, one study, n = 181; minocycline, one study, n = 544; monosodium L-glutamate, one study, n = 159; N-acetylcysteine (NAC), one study, n = 43; S47445, one study, n = 520; and sarcosine, one study, n = 30.

### Meta-analyses of overall cognitive function

**Figure 2:**
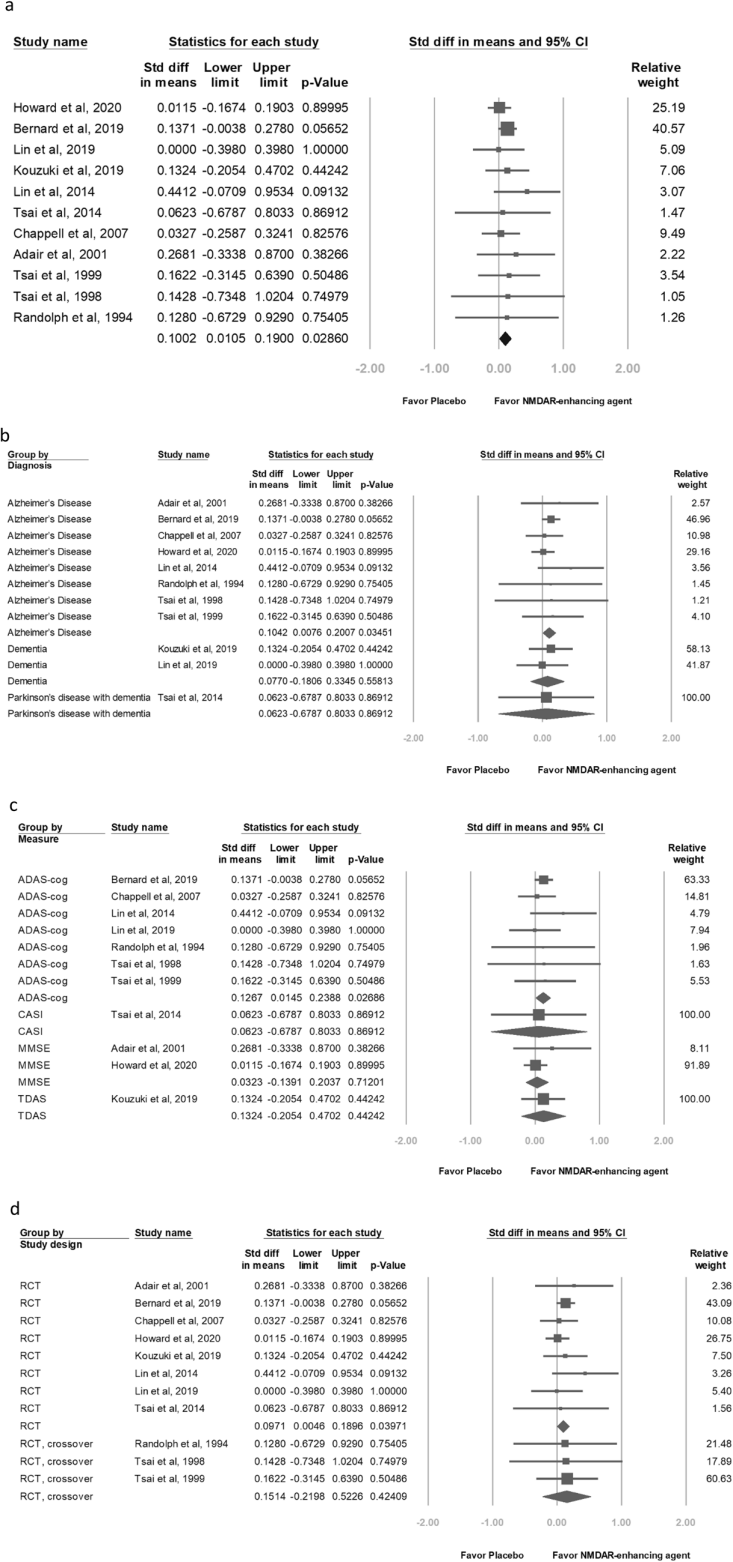

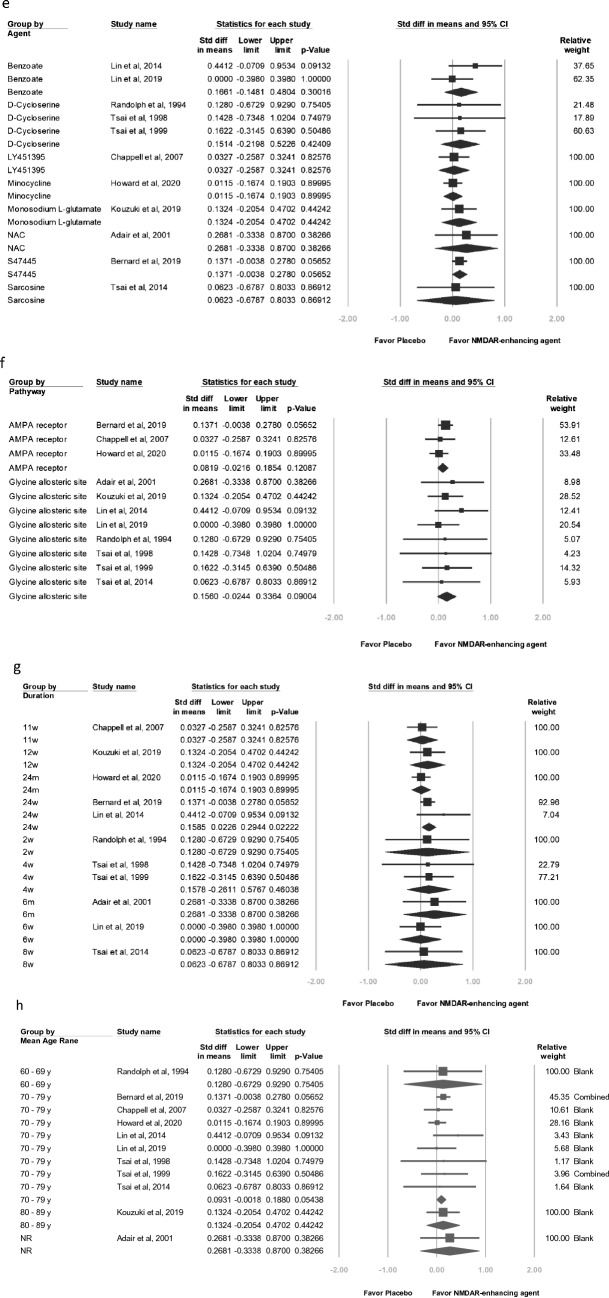
Meta-analyses of (**a**) overall cognitive function, (**b**) diagnosis of dementia groups, (**c**) cognitive measure groups, (**d**) study design groups, (**e**) NMDAR enhancing agent groups, (**f**) drug pathway in enhancing glutamatergic neurotransmission groups, (**g**) duration of therapy groups, and (**h**) age range groups.

Overall cognitive function data were reported in 11 studies^22–25,42–44,48,49,51,52^. Effects were conventionally categorized as small (SMD = 0.2), moderate (SMD = 0.5) or large (SMD = 0.8), with positive values indicating improvements in cognitive function. Overall, NMDAR enhancing modulators had a small positive significant effect on overall cognitive function among patients with dementia (SMD = 0.1002, 95% CI 0.0105–0.1900, *P* = 0.02860; Fig. 2a).

### Subgroup analyses

#### Diagnosis of dementia

This meta-analysis which pooled eight trials^22–25,43,44,48,51^ showed that the patients with AD revealed a significant but small effect (SMD = 0.1042, 95% CI 0.0076–0.2007, *P* = 0.03451; Fig. 2b).

#### Cognitive measure as the primary outcome

This meta-analysis which summarized seven trials^22–25,43,49,51^ that used the ADAS-cog as their cognitive measure reported small effects (SMD = 0.1267, 95% CI 0.0145–0.2388, *P* = 0.2686), whereas two trials^44,48^ that used the MMSE as their cognitive measure reported nonsignificant effects (SMD = 0.0323, 95% CI − 0.1391–0.2037, *P* = 0.71201; Fig. 2c).

### Study design

This meta-analysis which pooled eight RCTs^22,42–44,48–51^ reported significant positive effects (SMD = 0.0971, 95% CI 0.0046–0.1896, *P* = 0.03971). Three trials^23–25^ were crossover RCTs and discovered nonsignificant effects (SMD = 0.1514, 95% CI − 0.2198–0.5226, *P* = 0.42409; Fig. 2d).

### NMDAR enhancing agents

Furthermore, we performed a subgroup meta-analysis of the studies with add-on NMDAR enhancing agents to evaluate the overall cognitive effect. Nine NMDAR enhancing agents were investigated: benzoate, cycloserine, D-cycloserine, LY451395, minocycline, monosodium L-glutamate, NAC, S47445, and sarcosine. Subgroup meta-analyses for each NMDAR enhancing agent revealed small positive but nonsignificant differences in effect on overall cognitive function (Fig. 2e).

### Pathway through which the drugs enhance glutamatergic neurotransmission

#### Glycine allosteric site of NMDARs

A small positive but nonsignificant SMD was observed in the effect of drugs (benzoate, monosodium L-glutamate, sarcosine, NAC, and D-cycloserine) and placebo on overall cognition (SMD = 0.1560, 95% CI − 0.0244–0.3364, *P* = 0.09004; Fig. 2f).

### α-amino-3-hydroxy-5-methyl-4-isoxazole propionic acid (AMPA) receptors

A small positive but nonsignificant SMD was observed in the effects of drugs (minocycline, S47445, and LY451395) and placebo on overall cognition (SMD = 0.0819, 95% CI − 0.0216–0.1854, *P* = 0.12087; Fig. 2f).

### Treatment duration

This meta-analysis which summarized two trials^22,51^ with 24-week treatment duration discovered small positive significant effect (SMD = 0.1585, 95% CI 0.0226–0.2944, *P* = 0.02222; Fig. 2g).

### Patient age

This meta-analysis which pooled eight trials^22,23,25,42,43,48,49,51^ enrolling patients aged 70–79 years obtained nonsignificant small effect (SMD = 0.0931, 95% CI − 0.0018–0.1880, *P* = 0.05438; Fig. 2h).

## Meta-regression analyses of overall cognitive function

**Figure 3:**
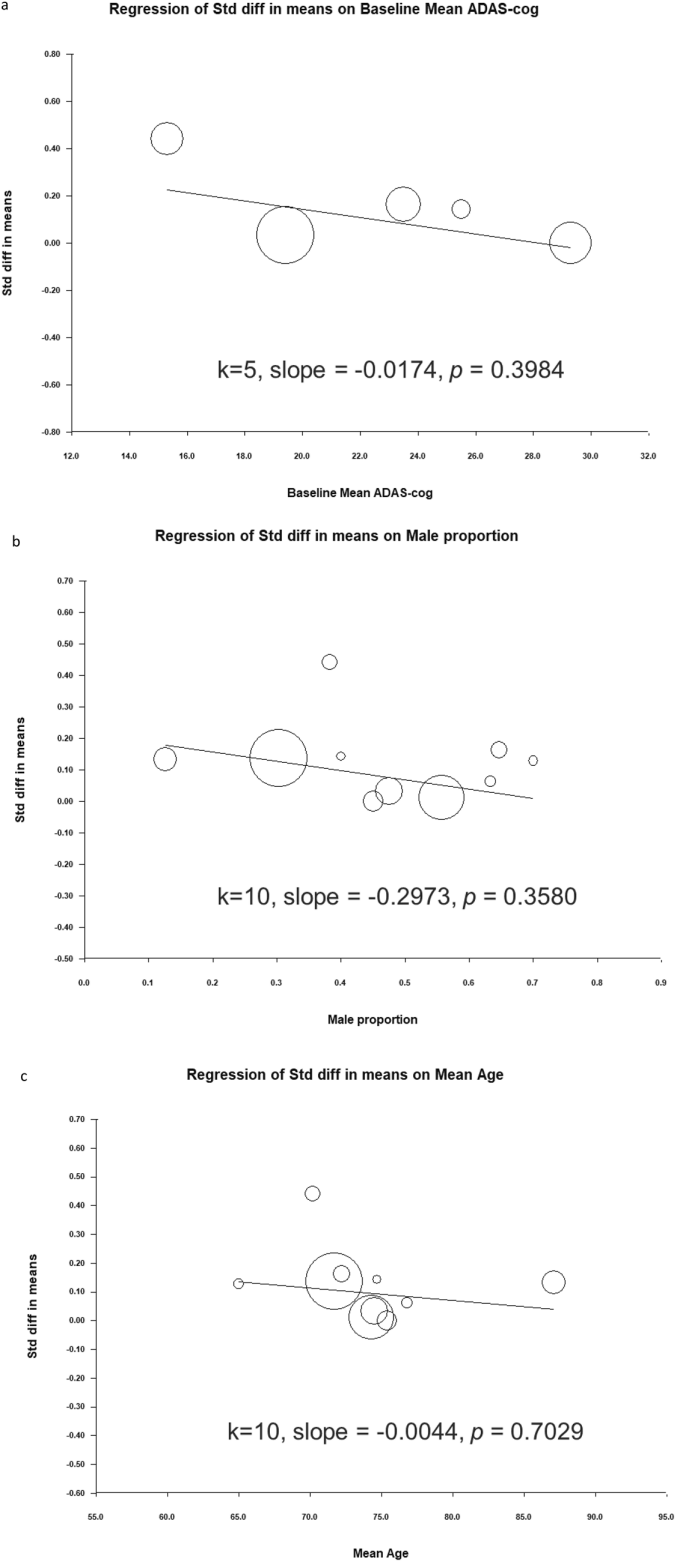
Meta-regression of effects of NMDAR enhancing agents on overall cognitive function in relation to (**a**) baseline mean ADAS-cog total score, (**b**) proportion of men, and (**c**) mean age.

Higher baseline mean ADAS-cog total score was associated but nonsignificant with higher proportion of men, higher mean age, and weaker effect of NMDAR modulators on overall cognitive function (baseline mean ADAS-cog score, slope =  − 0.0174, *P* = 0.3984; proportion of men, slope =  − 0.2973, *P* = 0.3580; age, slope =  − 0.0044, *P* = 0.7029; Fig. 3).

### Heterogeneity and publication bias

**Figure 4:**
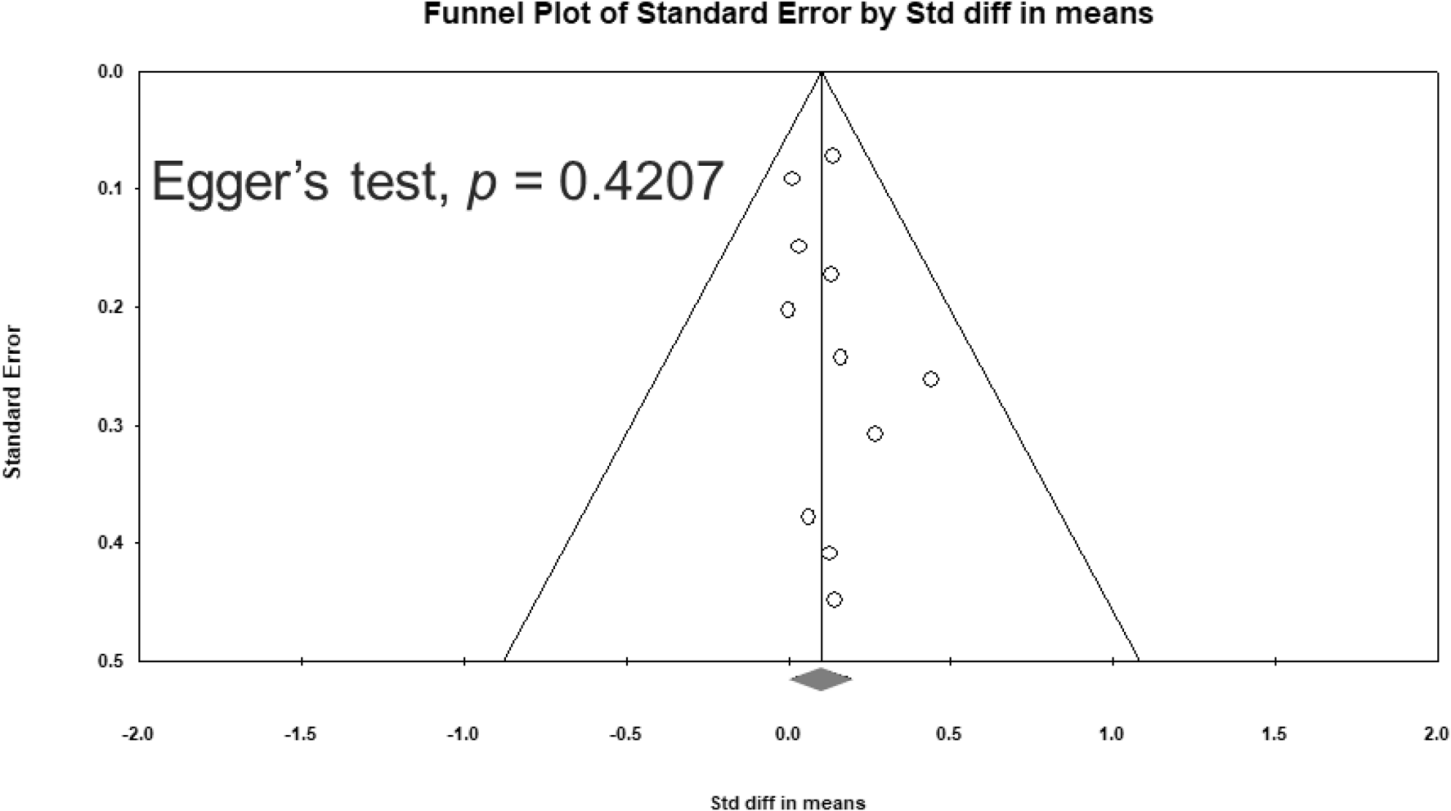
Funnel plots of overall cognitive function SMD.

No significant heterogeneity was observed in overall cognitive function among the studies (Q = 3.784, df = 10, I^2^ = 0.000%, *P* = 0.957). Egger’s test revealed no significant publication bias regarding the overall cognitive effects (*P* = 0.4207). The funnel plots for the effect sizes of overall cognitive function are displayed in Fig. 4.

### Sensitivity analysis

The positive effect of NMDAR enhancing agents on overall cognitive function became nonsignificant when the study by Bernard^51^ or Lin^22^ was removed.

#### Methodological quality of the included studies

Among the fourteen RCTs, the average Jadad score was 3.14 with a standard deviation (SD) of 0.36 (Supplementary Table S2).

## Discussion

To our knowledge, this is the first study to investigate the efficacy of NMDA enhancing agents on cognition among patients with dementia. The main findings of this analysis are that (1) NMDAR enhancing agents had small positive significant effects on overall cognitive function compared with placebo, (2) trials on patients with AD obtained small positive significant effects, (3) trials using the ADAS-cog as the primary outcome obtained small positive significant effects.

These findings accord with those of most related studies. Two of the studies reported significant improvement in overall cognitive function in patients with AD^22,23^. Lin and colleagues treated patients with mild AD with 250–750 mg/d of sodium benzoate or placebo for 24 weeks, and the patients treated with sodium benzoate displayed significantly greater improvement in ADAS-cog score (*P* = 0.0031)^22^. Tsai and colleagues treated 17 patients with AD with 50 or 100 mg/d of D-cycloserine or placebo for 4 weeks; the patients treated with D-cycloserine displayed significantly greater improvement in ADAS-cog score (placebo: 24.05 ± 10.99; 50 mg of D-cycloserine: 23.86 ± 10.19; 100 mg of D-cycloserine: 21.12 ± 8.82)^23^. Seven studies reported nonsignificant improvement in overall cognitive functions^24,25,43,44,48,50,51^. For example, Howard reported that patients treated with minocycline had mean MMSE score 0.1 points higher than those treated with placebo; however, the mean was nonsignificantly different (95% CI − 1.1–1.2; *P* = 0.90)^48^. Bernard reported that patients treated with S47445 had nonsignificant improvement in ADAS-cog score; the adjusted mean change in ADAS-cog total score from baseline to week 24 was 0.35 ± 5.53 in the placebo group, − 0.20 ± 4.91 in the 5 mg of S47445 group, − 0.74 ± 5.43 in the 15 mg of S47445 group, and − 0.05 ± 5.82 in the 50 mg of S47445 group^51^.

Subgroup analyses revealed that the meta-analysis which pooled eight trials enrolling patients with AD^22–25,43,44,48,51^ showed a small positive significant effect (SMD = 0.1042, 95% CI 0.0076–0.2007, *P* = 0.03451; Fig. 2b), while trials enrolling patients with non-AD dementia did not. This finding may imply that patients with AD may more benefit from NMDAR enhancing agents than patients with non-AD dementia. Lin and colleagues treated patients with mild AD receiving with 250–750 mg/d of sodium benzoate or placebo for 24 weeks; those treated with sodium benzoate displayed significantly greater improvement in ADAS-cog score (*P* = 0.0031)^22^. Their findings suggest that patients with mild AD may benefit more from NMDAR enhancing agents than patients with moderate to severe AD. However, in our meta-regression, no significant association between effect size and mean ADAS-cog was observed. Further studies are needed to investigate these findings.

Moreover, we noted that this meta-analysis summarizing seven trials^22,23,25,43,49,51^ in which the ADAS-cog was employed as the cognitive measure revealed small positive significant effects (SMD = 0.1267, 95% CI 0.0145–0.2388, *P* = 0.02686), whereas two trials^44,48^ using the MMSE reported nonsignificant effects (SMD = 0.0323, 95% CI − 0.1391–0.2037, *P* = 0.71201; Fig. 2c). The MMSE and ADAS-cog are most common cognitive measures in these trials. The MMSE is often employed to screen for dementia and measure cognitive impairment in older adults^35^. The MMSE total score ranges from 0 to 30 (highest to lowest level of cognitive impairment) while the ADAS-cog consists of 11 tasks, with a total score ranging from 0 to 70 (lowest to highest level of cognitive impairment)^53^. MMSE and ADAS-cog have shown to be relatively psychometrically poor at detecting small changes^54–56^. Therefore, researchers may use more sensitive instruments like Addenbrooke's Cognitive Examination^57^ or Montreal Cognitive Assessment^58,59^ to explore minor changes in cognition in the early stages of dementia receiving NMDAR enhancing agents.

The mechanism of the positive effect of NMDAR enhancing agents on cognition remains unclear. Meta-analytic studies have investigated NMDAR enhancing agents in patients with schizophrenia. Tsai and colleagues reported that the overall effect of NMDAR enhancing agents (glycine, D-serine, and sarcosine) on cognitive symptoms was 0.28 (95% CI 0.10–0.47, *P* = 0.002, 13 studies, n = 485)^29^, whereas two other meta-analytic studies did not report a significant effect on overall cognition in schizophrenia^26,28^. An updated meta-analysis enrolling 25 trials and 1951 participants revealed no significant effect of NMDA-enhancing agents on overall cognition. However, subgroup analysis suggested that NMDAR-enhancing agents may benefit young patients with schizophrenia, and NAC may have effect on working memory^27^. Human studies have reported an age-related decrease in the density of NMDARs in the cerebral cortex and hippocampus^19^. NMDAR activation improves memory function^12^. Several trials with NMDAR enhancing agents, such as benzoate and D-cycloserine, have reported promising effects, especially in early-stage AD^22,23^.

### Side effects

The side effects were mostly mild and relieved after discontinuing the agents. The side effect rate of NMDAR enhancing agents ranged from 0 to 61.1%. Minocycline was reported side effects including dermatologic symptoms (hyperpigmentation, photosensitivity, rash), gastrointestinal symptoms (diarrhea, nausea, sore mouth, vomiting), neurologic symptoms (headache, visual or auditory disturbances, dizziness), infections (oral or genital candidiasis, vaginitis, anal irritation, bacterial enteritis, staphylococcal, or Clostridium difficile). Six skin toxic effects were considered severe^48^. The most common adverse effects of the study with AMPA modulator S47445 are nasopharyngitis, blood creatine phosphokinase increased, diarrhea, fall, headache, type 2 diabetes mellitus, abdominal pain^51^. D-cycloserine was reported no side effects^23,25^. Detailed information about the adverse events addressed in the included studies is summarized in Supplementary Table S2. The attrition rate ranged from 0 to 0.49. The effect size in this exploratory meta-analysis was very small. Most side effects were mild. The benefit may weigh up against the side effects.

### Implication

Cognitive impairment is a critical problem in central nerve disease such as dementia. AD is the leading cause of dementia. In 2010, the number of patients with AD was estimated at 36.6 million and is projected to double every 20 years^2^. Dementia is a considerable economic burden on both patients and society^6^. However, understanding of the complex mechanisms and treatment response remains unsatisfactory^3^. Therefore, developing alternative therapeutic approaches is crucial. Our meta-analysis revealed a positive and significant effect of NMDAR positive modulators on overall cognition among patients with dementia. Further trials with better design and larger samples are needed to explore NMDAR enhancing agents in MCI or early-phase dementia.

### Strength

The present study had several strengths. First, this is the first meta-analytic study to evaluate the treatment effect of NMDAR positive modulators among patients with dementia. Second, nine compounds were included in this meta-analysis. Third, we noted that NMDAR enhancing agents may have a small positive significant effect on overall cognitive function compared with placebo.

### Limitations

There are several limitations in the present study. First, this is an exploratory systematic review and meta-analysis of randomized controlled trials. We could observe related phenomena but not clarify the underlying pathophysiology because of the methodological limitations of meta-analyses. Therefore, caution should be exercised when drawing conclusions. Second, the number of participants was small in some included trials. Third, some trials were conducted over a short period (less than 6 months). Therefore, the long-term cognitive effects of NMDAR enhancing agents and the persistent effects after treatment remain unclear. Kouzuki and colleagues reported improvement in the Touch Panel-type Dementia Assessment Scale total score during a 4-week follow-up assessment^50^. Fourth, the trials used different standard cognitive tests. Fifth, nine NMDAR enhancing agents were investigated. These agents may have different mechanisms of action involving the *N*-methyl-d-aspartate (NMDA) system. Further studies are needed to explore the relationships between neurocognitive effects and specific NMDAR enhancing mechanisms. Some modulators may be involved in mechanisms other than the NMDA system. Sixth, the effects of the stage of dementia and concomitant antidementia drugs remain unclear.

## Conclusion

The findings from this meta-analysis indicated that NMDAR enhancing agents showed a very small positive effect on overall cognitive function in patients with dementia. Further studies with a larger sample are warranted to explore the role of the NMDA system on specific cognitive domains in subgroups of patients with early-stage dementia.

## Supplementary Information

Supplementary Information 1.

Supplementary Information 2.
