# Supplementary material for: Effect of N-methyl-d-aspartate receptor enhancing agents on cognition in dementia: an exploratory systematic review and meta-analysis of randomized controlled trials
Source: Sci Rep. 2021 Nov 26;11:22996. doi: 10.1038/s41598-021-02040-5 (PMC8626464; doi:10.1038/s41598-021-02040-5)
Supplement: Supplementary file 2 — Supplementary Information 2. [file 41598_2021_2040_MOESM2_ESM.pdf]

Supplementary Table S2. Summary of the characteristics of studies included in the meta-analysis.

Study (first author, year)	Dementia severity	Anti-dementia drugs	Adjunctive or combined treatments	Attrition rates	Quality assessment (Jadad score)	Illness duration	Education	Side effects	Side effect rate
**Howard et al, 2020**	mild<br>(MMSE 24 to 30)	NR	NR	0.49	4	23.59±18.04 months	NR	Dermatologic symptoms (hyperpigmentation, photosensitivity, rash), gastrointestinal symptoms (diarrhea, nausea, sore mouth, vomiting), neurologic symptoms (headache, visual or auditory disturbances, dizziness), infections (oral or genital candidiasis, vaginitis, anal irritation, bacterial enteritis, staphylococcal, or Clostridium difficile)	serious adverse events: placebo group (58.7%, 105/179 patients) v.s. Minocycline group (40.3%, 147/365 patients)
**Bernard et al, 2019**	mild to moderate<br>(MMSE 15 to 24)	Donepezil	none	0.09	4	3.60±2.20 years	NR	Nasopharyngitis, blood creatine phosphokinase increased, diarrhea, fall, headache, type 2 diabetes mellitus, abdominal pain upper	placebo group (50.4%, 65/129 patients) v.s. S47445 group (45.3%, 177/391 patients)
**Lin et al, 2019**	mild to severe<br>(MMSE 5 to 26)	Donepezil<br>Rivastigmine<br>Galantamine<br>Memantine	Quetiapine<br>Risperidone<br>Olanzapine<br>Aripiprazole<br>Sulpiride	0.13	3	2.05 years	4.70±4.10 years	One benzoate recipient reported mild and brief polyuria; one placebo receiver experienced moderate tension sensation.	placebo group (2.1%, 1/48 patients) v.s. benzoate group (2.0%, 1/49 patients)
**Kouzuki et al, 2019**	moderate	NR	NR	0.14	3	NR	NR	NR	NR
**Lin et al, 2014**	mild<br>(CDR 0.5 to 1)	Donepezil<br>Rivastigmine<br>Galantamine	none	0.1	3	13.90±16.79 months	6.70±4.96 years	Only one patient in the placebo group reported dizziness at week 16.	placebo group (3.3%, 1/30 patients) v.s. benzoate group (0%, 0/30 patients)
**Tsai et al, 2014**	mild to moderate	NR	NR	0.17	3	2.35±1.83 years	7.40±5.54 years	dizziness, hypersomnia	placebo group (6.7%, 1/15 patients) v.s. Sarcosine group (6.7%, 1/15 patients)
**Chappell et al, 2007**	mild to moderate<br>(MMSE 14 to 18 or 19 to 24)	none	SSRI,<br>trazodone,<br>lorazepam	0.08	5	NR	NR	nausea and insomnia	placebo group (61.5%, 56/91 patients) v.s. LY451395 group (61.1%, 55/90 patients)
**Adair et al, 2001**	NR	none	none	0.09	3	NR	NR	Fatigue, headache, appetite change, mood change, muscle ache/cramp, diarrhea, dizziness, arthralgia, abnormal dreams, rash	placebo group (0 to 16.7%) v.s. N-acetylcysteine group (4.3 to 26.1%)
**Tsai et al, 1999**	NR	NR	NR	0	3	NR	NR	none	0%

**Tsai et al, 1998**	mild to moderate (MMSE 10 to 25)	NR	NR	0	3	NR	NR	none	0%
**Schwartz et al, 1996**	mild to moderate (MMSE 12 to 24)	NR	NR	0.16	3	NR	13.22±2.89 years	NR	NR
**Mohr et al, 1995**	mild to severe (MMSE 12 to 24)	NR	NR	NR	3	NR	NR	upper respiratory tract infections, headache, accidental injury, agitation, arthritis, and confusion	NR
**Fakouhi et al, 1995**	mild to moderate (MMSE 12 to 27)	NR	NR	0.34	3	NR	12.60±3.00 years	upper respiratory tract infections, headache, accidental injury, agitation, arthritis, and confusion	NR
**Randolph et al, 1994**	mild to moderate (MMSE 15 to 25)	NR	NR	0	3	NR	NR	transient agitated-confusional state	placebo group (0%, 0/12) v.s. D-Cycloserine group (8.3%, 1/12)

Abbreviations: CDR, Clinical Dementia Rating; MMSE, Mini-Mental Status Examination; NR, no reported.

The MMSE ranges were 0-10 for severe dementia, 11-20 for moderate, 21-25 for mild, 26-29 for questionable, and 30 for no.
